# Job mismatches and their implications for the career development of vocational graduates

**DOI:** 10.1186/s40461-026-00207-w

**Published:** 2026-04-22

**Authors:** Laura Eberlein, Dimitris Pavlopoulos, Wendy Smits

**Affiliations:** 1 Vrije Universiteit Amsterdam, Amsterdam, Netherlands; 2 Research Centre for Education and the Labour Market (ROA), Maastricht University, Maastricht, Netherlands; 3 Centraal Bureau voor de Statistiek, Heerlen, Netherlands

## Abstract

The transition from vocational education into the labour market is a key indicator of how effectively VET systems prepare graduates for employment. A central concern for school-based and work-based programmes alike is whether education-job mismatches undermine vocational adjustment and early career development. This study examines how mismatch at labour-market entry is associated with graduates’ subsequent five-year employment trajectories in the Netherlands. We combine the 2016 Dutch School Leaver Survey with monthly register data from 2015–2020 for 10,364 school leavers. Using a Mixture Hidden Markov Model (MHMM), we identify five early-career trajectories that capture variation in employment stability, contract quality and progression. The results show that education-job mismatches at the beginning of the career are associated with a higher probability of following the Volatility & Entrapment trajectory, which is characterised by temporary contracts, limited progression and high turnover. Dual mismatches (vertical and horizontal combined) are linked to a notably lower likelihood of upward mobility, suggesting persistent vocational disadvantages that extend beyond the point of entry. Importantly, the findings reveal differences in vocational resilience across programme types: The negative associations of mismatch are more pronounced for school-based graduates. While work-based graduates show greater resilience to single mismatches, any type of mismatch is associated with a lower probability of following a successful early career for school-based graduates.

**Supplementary Information:**

The online version contains supplementary material available at 10.1186/s40461-026-00207-w.

## Introduction

The transition from education to work is a pivotal stage in shaping career trajectories. Establishing secure and high-quality employment during this initial phase is crucial, as the first job strongly influences subsequent vocational outcomes and long-term career success. A ’bad entry’ into the labour market–such as experiencing an education-job mismatch–can have lasting negative consequences (Schmelzer and Schneider 2020; Steijn et al. 2006). Given the inherent uncertainty of this transition, young people may enter jobs that do not align with their field of study or qualification level, which can hinder their integration into the labour market and affect subsequent job quality.

Previous literature on education-job mismatches indicates that working in a job below one’s level of education is associated with lower wages (Béduwé and Giret 2011; Levels et al. 2014a; Arranz and García-Serrano 2025), reduced job satisfaction (Verhaest and Verhofstadt 2016), and higher turnover rates (Verhaest and Omey 2006). Penalties for such vertical mismatches appear to be particularly severe for individuals with occupation-specific training, such as vocational-education graduates (Ryan and Sinning 2011).

In addition to vertical mismatches, horizontal mismatches–where the job does not correspond to the field of study–are also important for young workers. Although research on horizontal mismatches is growing, the findings remain inconclusive. Some studies suggest that horizontally mismatched workers suffer significant wage penalties (see Somers et al. (2019) for an overview) and reductions in occupational status (Wolbers 2003), while others report only minor or insignificant effects on wages and job satisfaction (Schweri et al. 2020; Béduwé and Giret 2011).

The inconclusiveness of these findings may be indicative of differences in institutional contexts, particularly the strength of the link between educational credentials and the labour market. In systems with a strong link, the earnings premium for a good occupational match is high, but so is the penalty for a mismatch (Bol et al. 2019). At the same time, institutional explanations may overlook within-country heterogeneity, as even within a single system, educational credentials can be institutionalised differently, affecting both the content of programmes and their labour market outcomes.

Another factor that may contribute to the heterogeneity of findings in the literature is the widespread practice of conceptualising horizontal and vertical mismatches as distinct phenomena, despite evidence that these two forms of mismatch frequently correlate in practice (Schweri et al. 2020). The few studies that investigate their combined effects (see, for example, Béduwé and Giret 2011; Kim et al. 2012; Robst 2008, for noteworthy exceptions) suggest that the experience of both mismatch types in combination has a particularly detrimental effect on wages and job satisfaction. Furthermore, failing to account for vertical mismatch when estimating wage effects arising from horizontal mismatch may result in biased estimates, as wage penalties may be partly a reflection of downward occupational mobility as opposed to field mismatch itself (Kim et al. 2012). This underscores the necessity for an integrated analysis of both mismatch types and their associations with career trajectories.

This study examines the relationship between vertical, horizontal, and combined education-job mismatches and the early careers of school-based and workplace-based vocational graduates in the Netherlands. The Netherlands provides an ideal context for this study. At the upper secondary level of Dutch education, numerous vocational specialisations are offered, either through full-time vocational education and training (VET) schools or work-based apprenticeships. These programmes differ notably in their emphasis on theoretical versus practical, occupation-specific skills.

Although the incidence of both vertical and horizontal mismatches in the Dutch labour market has been well-documented, little is known about their implications for career trajectories. Most research has focused on labour market entries during recessions, neglecting broader career implications over time (Wolbers 2014). Moreover, while the literature often prioritises vertical mismatches, evidence suggests that horizontal mismatches are more common in vocationally oriented systems, like that of the Netherlands (Levels et al. 2014b). In such systems, the signalling power of vocational qualifications may reduce employer uncertainty about candidates’ skills, making horizontal mismatches particularly consequential for career mobility.

Our study advances the theoretical and empirical dimensions of the mismatch literature in three important ways. First, we examine both the separate and combined implications of vertical and horizontal educational mismatch at labour market entry. Second, we adopt a dynamic perspective by analysing early career trajectories rather than single labour-market outcomes. Whereas most studies examine the relationship between mismatch and outcomes such as wages or employment status at a single point in time, we investigate how mismatch is associated with graduates’ employment trajectories over the first five years after graduation. Third, we apply a multidimensional trajectory approach to construct a longitudinal typology of employment quality. By simultaneously capturing employment stability, contract quality, working hours, wages, and cumulative periods of unemployment, this approach provides a more comprehensive account of how education–job mismatches relate to the successful labour-market integration of young vocational graduates.

We employ a Mixture Hidden Markov Model (MHMM) on a unique dataset linking the 2016 Dutch School Leaver Survey, which includes information about education-job matching, with monthly register data on employment and wages from Statistics Netherlands (2015–2020). The MHMM allows us to summarise key dimensions of labour market integration, including employment, contract type, wage growth and working hours, into a latent measure of employment quality, while simultaneously identifying trajectory types that capture changes in this latent variable over time. A multinomial logistic regression assesses the relationship between vertical, horizontal, and combined mismatches and the identified trajectory types across different vocational tracks. While our longitudinal design and unique register-linked data provide a detailed account of early career pathways, we interpret these patterns as descriptive associations reflecting potential selection and equilibrium labour-market sorting rather than direct causal effects.

## The Dutch VET system

To understand the relationship between mismatches and VET graduates’ career trajectories, it is important to first examine the Dutch context. The Netherlands has a well-funded VET system that aligns training standards with occupational requirements, fostering strong linkages between education and the labour market.

In upper-secondary vocational education (in Dutch: *Middelbaar Beroeps Onderwijs, or MBO*), students can choose between two tracks leading to formally equivalent qualifications: the work-based ’apprenticeship’ track (BBL) and the school-based track (BOL). In 2014/15, approximately 54% of graduates completed upper-secondary education, with 70% following the BOL track and 30% the BBL track (CBS 2023). Although both tracks combine classroom learning with work-based training, they differ significantly in their emphasis. The school-based track requires at least 20% of study time to be allocated to practical work, typically averaging 29% (Vrieze et al. 2009). In contrast, apprentices in the work-based track spend at least 60% of their training in a firm, typically working four days a week and attending a regional training centre one day. The fundamental distinction lies in the predominant nature of the training environment: school-based training has a greater emphasis on theoretical learning, while work-based training is predominantly focused on practical application, with apprentices receiving an employment contract with their training firm and wage payments based on industry-level agreements.

VET programmes encompass four main fields: health, welfare, culture, and sport; engineering and construction; environmental studies and food; and economics, business, ICT, and hospitality. Training levels range from entry-level (1 year, ISCED 254) to basic (2 years, ISCED 353), professional (3 years, ISCED 353), or middle-management training (4 years, ISCED 354), with the latter allowing graduates to pursue tertiary professional education. Levels 2 to 4 provide a *starting qualification* and prepare students for immediate entry into the labour market (or further studies). Level 1 training primarily serves as a foundation for further education, targeting students under 18 who are required by law to continue education until they obtain an entry-level qualification.

The Dutch VET system is designed to align closely with labour market demands through structured collaboration between education providers and industry representatives. Sector-specific vocational bodies, comprising both employer and employee representatives, shape qualification structures, oversee apprenticeship placements, and set criteria for firms that wish to train apprentices. This ensures that students are equipped with relevant, occupation-specific skills. This alignment is further strengthened by a National Qualifications Framework, which is continuously updated with input from these sectoral bodies. The delivery of these programmes is the responsibility of regional VET colleges, which maintain a degree of autonomy over their study programmes, examinations and budgets, allowing them to adapt to regional needs within the national framework. However, this autonomy comes with an expectation: VET colleges are expected to offer programmes only if they meet labour-market needs, ensuring that graduates are prepared for the opportunities available in the region. Despite these institutional efforts to ensure a seamless transition, education–job mismatches remain a persistent feature of the Dutch labour market.

## Theoretical background

### Education-job (Mis)matches and labour market outcomes

Career mobility theory and matching models suggest that workers may enter a position that initially does not align with their educational background if it facilitates the acquisition of skills that can be leveraged to secure a higher-level position inside or outside the firm at a later stage (Sicherman and Galor 1990). Job mismatches are thus regarded as a transitional phenomenon, potentially serving as a ’stepping stone’ to higher-quality employment (Büchel and Mertens 2004; Arranz and García-Serrano 2024).

In contrast, assignment theory anticipates negative consequences of education–job mismatches for early career outcomes. According to this perspective, wages and productivity are determined by the quality of the match between workers’ skills and job requirements (Sattinger 1993). Importantly, education-job mismatch represents an equilibrium outcome of the matching process between graduates and employers rather than a purely individual choice (Jovanovic 1979). Within VET systems, where training is highly occupation-specific, employers play a central role in screening and allocating workers to available positions. Mismatch may therefore emerge from structural constraints and local demand conditions, such as limited vacancies or firm-level rationing, rather than worker preferences alone. When young workers enter positions outside their field of training or below their qualification level, they cannot utilise their occupation-specific knowledge acquired during education. Instead, they must invest significant effort into learning new, job-specific skills, leading to adjustment costs and lower initial productivity (Montt 2017). Consequently, mismatched workers experience lower starting wages and slower wage progression compared to well-matched peers. Assignment theory therefore predicts wage penalties associated with vertical and horizontal mismatches.

Moreover, according to signalling theory, horizontal and vertical mismatches early in a career may function as a negative signal to future employers, who may perceive them as indicators of lower skill levels or adaptability (Mavromaras and McGuinness 2012; Thurow 1975). Employers regard credentials as reliable indicators of occupational skills, yet they may lack additional information about an applicant’s potential. Consequently, an initial job mismatch may signal low productivity, a perception that is likely amplified in cases where both vertical and horizontal mismatches coincide. Education-job mismatches have thus the potential to restrict career opportunities, potentially trapping workers in lower-quality jobs, because they function as a marker for persistent labour-market sorting (Gibbons and Waldman 1999). Within occupational labour markets, such as the Dutch VET system, where credentials are closely linked to specific job roles, these signalling effects may be especially pronounced and can have long-lasting consequences for earnings and employment stability.

There is little empirical evidence to support the career mobility theory, which posits that job mismatches at the beginning of the career are temporary and do not have lasting negative consequences (Frei and Sousa-Poza 2012; Vogtenhuber 2014). A recent study by Arranz and García-Serrano (2025) provides partial support to this perspective. Their findings suggest that, at the time of entry into the labour market, overeducated workers in Spain earn substantially less than their well-matched peers. However, those who change employers over time are able to close this wage gap, consistent with the career mobility perspective, whereas those who remain in mismatched positions continue to face persistent wage penalties (Arranz and García-Serrano 2025). Nevertheless, most empirical evidence on vertical mismatches indicates that accepting a job for which one is overeducated can become a trap, delaying transitions to better-suited roles and resulting in slower wage growth (Baert et al. 2013; Büchel and Mertens 2004; Scherer 2004; Schmelzer and Schneider 2020; Verhaest and Van der Velden 2013). This adverse effect tends to be more pronounced for vocational graduates than for university graduates (Ryan and Sinning 2011), suggesting that VET graduates may find it particularly difficult to transition out of low-level positions once they experience a mismatch.

With regard to horizontal mismatches, the evidence is more inconclusive. Previous findings show that, in the Netherlands, a good match between the field of study and the labour market is positively associated with wages (Van de Werfhorst 2002), while horizontal mismatches are associated with lower occupational-status attainment and higher turnover rates in several European countries (Wolbers 2003). In a review article, Somers et al. (2019) confirm that horizontal mismatches generally result in a wage penalty and lower job satisfaction, a finding that is consistent with assignment theory. In contrast, Béduwé and Giret (2011) find that the widespread mismatch for vocational graduates in the French labour market does not lead to significant wage penalties. However, they associate horizontal mismatches with higher job dissatisfaction and a greater desire to seek alternative employment, even in highly qualified, permanent, and reasonably paid positions. Similarly, Schweri et al. (2020), find only negligible wage penalties for horizontal mismatches among both vocational and general education graduates, suggesting that vocational education may be more transferable than often assumed.

Education-job mismatches may also be linked to early career instability. Previous research has demonstrated that graduates in well-matched roles are more likely to obtain permanent contracts (Wolbers 2003), as employers have a vested interest in retaining such workers. Such arrangements are also beneficial for workers, who enjoy security in the form of permanent employment, improved career prospects and, consequently, greater opportunities for upward mobility (Fouarge et al. 2012). Conversely, employers may be reluctant to offer permanent positions to workers whose formal credentials do not match job requirements, leading to job insecurity, lower wages and less firm-specific training. This may also result in lower job dissatisfaction (Verhaest and Verhofstadt 2016), which, in turn, may drive individuals to seek better job matches, potentially leading to more unstable career trajectories. Based on this reasoning, we hypothesise that graduates with a non-matching job are more likely to follow career trajectories characterised by frequent job transitions, temporary positions, low wages and unemployment compared to those with a matching job *(Hypothesis 1a)*.

Furthermore, a full mismatch – encompassing both a horizontal and vertical mismatch – may be associated with particularly disadvantageous career trajectories of young graduates. Overeducated job starters who also work in an occupational field that does not match their formal qualifications are likely to experience a rapid depreciation of their human capital. Moreover, these individuals fail to accumulate any significant firm-specific human capital in their occupational field, thereby making it increasingly challenging for them to transition back to roles that are aligned with their qualifications. Furthermore, the stigma associated with vertical and horizontal mismatch is likely to be exacerbated when both types of mismatch coincide, resulting in adverse career outcomes. Therefore, we expect the relationship of *Hypothesis 1a* to be stronger for a full mismatch *(Hypothesis 1b)*.

### Expected differences between work-based and school-based graduates

While we expect that vertical, horizontal and full mismatches are negatively associated with career trajectories of vocational graduates in general, the extent of this impact may vary depending on the type of vocational education. Despite that both workplace-based and school-based vocational education lead to the same formal qualifications, they differ significantly in terms of the nature and specificity of the skills they impart, which may influence how graduates experience and navigate job mismatches.

Workplace-based programmes emphasise practical, occupation-specific skills acquired through extensive on-the-job training. Apprentices acquire skills tailored to the needs and practices of their firms (Coenen et al. 2015). If a worker leaves the firm, the firm loses part of the return on its training investment, which is why firms have an incentive to retain trained employees to recoup their investment (Hashimoto 1981). Although some apprenticeship-acquired skills are transferable to other firms using similar technologies, their specificity increases the likelihood of retention within the training company, thereby minimising the risk of mismatches. In addition, work-based training also serves as an extended screening period, enabling employers to assess workers’ skills and suitability. Graduates hired by their training firms can immediately apply firm-specific skills, reducing the need for additional training or screening. Thus, workplace-based graduates are less likely to experience vertical and horizontal mismatches compared to their school-based counterparts *(Hypothesis 2)*.

However, the initial advantage of work-based graduates can readily transform into a disadvantage if they are unable to obtain a vertical and horizontal match. The inability to secure a matching job may convey a negative signal to future employers, potentially impeding career advancement (Shi and Wang 2022). This ’bad signal’ is likely to be more pronounced for work-based graduates. If these graduates are unable to secure employment in their training firm or a matching job with a similar employer, employers may perceive this as an indication of low competence (Pedulla 2016) and a lack of ability to capitalise on the practical training they have received. Such perceptions can significantly reduce employment chances, potentially trapping workers in a cycle of low-quality jobs. In addition, when these graduates face a job mismatch, the firm-specific skills they acquired through training are rendered obsolete. In contrast, while mismatches convey some negative signalling for school-based graduates, negative consequences for their career trajectories may be less severe. Employers generally expect a learning curve and anticipate that school-based graduates will require additional on-the-job training to become fully productive. Consequently, an early job mismatch is less likely to be seen as a sign of poor performance or ability. Therefore, we argue that workplace-based graduates, when confronted with a vertical, horizontal or full mismatch, face greater difficulty in recovering from such early career setbacks due to the more pronounced negative signalling associated with their mismatched employment histories and the limited transferability of their firm-specific skills. Therefore, the negative association between job mismatches and career trajectories is more pronounced for workplace-based VET graduates compared to school-based VET graduates *(Hypothesis 3)*.

## Empirical strategy

### Data and sample

A unique dataset is used that combines individual-level information from registers and survey data. We use the 2016 Dutch School Leavers Survey (SVO, *in Dutch: Schoolverlatersonderzoek 2016*), an annual, nationally representative survey conducted by Statistics Netherlands targeting secondary-school leavers. School leavers who completed their education during the 2014/2015 school year, were interviewed in autumn 2016, approximately one year after leaving education. The response rate for upper secondary VET graduates, levels 2–4 (equivalent to ISCED 3) was 26.5%. As part of the survey, school leavers were asked to provide information about their education and training as well as their transition into the labour market. The questionnaire incorporated items asking about the match between their work and field of education, allowing the identification of vertical and/or horizontal mismatches at the beginning of their career.

To study the careers of school leavers over a longer period, we linked the survey to individual-level register data for 2015-2020 from Statistics Netherlands (Bakker et al. 2014). The register data comprises monthly information on employment, wages and working hours, allowing for the longitudinal tracking of school leavers over a period of 65 months following their departure from education.

The sample has been restricted to school leavers from VET levels 2,3 and 4. We excluded school leavers who continued education immediately after completing their previous degree, and those older than 27 years at the time of leaving education to ensure that our sample contains individuals who enter the labour market for the first time^1^. Furthermore, we excluded individuals observed for less than 75% of the observation period (2.7% of the sample). This includes individuals who have, for example, relocated abroad and are, therefore, no longer part of the register. Individuals who are inactive but still registered in the Netherlands are included in the sample. Furthermore, the sample was restricted to individuals who had experienced at least one employment episode during the observation period. This restriction was applied because individuals who never entered employment after leaving education^2^ lack information on education–job mismatch, which constitutes the key variable of the analysis. Table 1 of the supplementary material presents a comprehensive overview of the inclusion criteria and the corresponding change in case numbers. The resulting analytical sample for constructing employment trajectories comprises 684,588 person-month observations nested in 10,555 individuals. To investigate the relationship between mismatch and the resulting employment trajectories, the sample size is reduced to 10,364, as records containing missing values for any of the independent variables are excluded. Out of these individuals, 2,517 were enrolled in the work-based track, while 7,847 were enrolled in the school-based track.

### Typology of employment trajectories

Our dependent variable is a typology of employment trajectories, which is estimated using a Mixture Hidden Markov Model. This approach enables the simultaneous assessment of diverse aspects of labour market integration and success by combining outcomes on wages, employment contract and working hours into the latent variable of Employment Quality (EQ), which is then modelled over time to identify distinct employment trajectories. While numerous definitions for EQ exist in the literature (see Green (2013) for an overview), there is some consistency in the characteristics considered to be crucial for the well-being of workers and the successful integration of young people into labour market. These characteristics can be broadly classified as indicators of earnings and security (Berloffa et al. 2019). Therefore, our latent EQ variable is approximated with three indicators: employment status, income and working hours.

The employment status indicator differentiates between permanent, fixed-term, temporary-agency, and on-call contracts, in addition to distinguishing those who are self-employed or non-employed. Multiple job holders^3^ are classified according to their primary activity in the labour market, defined by their main source of income. Because information on self-employment is derived from annual tax statements, we cannot determine the precise number of months spent in self-employment within a year. Therefore, individuals are classified as self-employed for a given month if their average monthly income from self-employment exceeds their income from any other source.

Income is defined as total gross monthly earnings from wages and social benefits, adjusted for inflation and categorised into quintiles. For the self-employed, we divided annual earnings by 12 to obtain monthly earnings, covering all forms of self-employed activity. Including benefits reflects the Dutch institutional context, where periods of unemployment or early-career instability are often partially compensated through social transfers. Therefore, income captures the effective economic resources and security rather than pure wage performance. Importantly, because employment status is modelled as a separate indicator in the latent employment quality construct, individuals receiving benefits during non-employment spells remain clearly distinguishable from those in stable employment. This ensures that the latent trajectories capture the multifaceted nature of labour-market attachment beyond simple earnings.

Finally, weekly working hours are aggregated in five categories: no hours, marginal part-time (less than 12 hours), short part-time (12-<20 hours), substantial part-time (20–35 hours) and full-time (>35 hours).

### Independent variables

#### Education-job mismatch

The two main independent variables are education-job mismatch, and the type of vocational training attended. Concerning the latter, we distinguish between school-based and workplace-based vocational training. Education–job mismatch is measured using respondents’ self-assessments collected in the SVO survey, which capture information on the level and field of education required by their employer to perform their job. In the literature, mismatch can be operationalised using either objective measures, typically based on expert evaluations or calculated modal educational values within occupations, or subjective measures that rely on workers’ self-assessments of how their education corresponds to the requirements of their job (Leuven and Oosterbeek 2011). Subjective assessments have the advantage of ’obtaining information from the source closest to the actual job situation, taking account of all specific circumstances’ ((Hartog and Oosterbeek 1988), p.186). Indeed, Van Der Velden and van Smoorenburg (1997) found that, while job-analyst methods tend to overestimate overeducation, no such bias was evident in worker self-reports. Nevertheless, self-reports are susceptible to reporting biases, including social desirability and imperfect information regarding true job requirements. Ultimately, as is common in applied research, the choice of measurement approach is constrained by data availability (Arranz and García-Serrano 2025). Since the SVO data provide only subjective indicators, we rely on these self-reported measures while acknowledging their limitations and interpreting the results with appropriate caution.

Furthermore, our measure captures credential mismatch–the formal misalignment between attained degrees and job requirements–rather than direct multidimensional skill mismatch or actual skill use (Lise and Postel-Vinay 2020; Pellizzari and Fichen 2017; Guvenen et al. 2020). While recent research increasingly uses proficiency measures to distinguish between what workers *know* versus what they *do* (Woessmann 2024; Kawaguchi and Toriyabe 2022), our focus on credentials reflects the primary policy-relevant indicator in the highly institutionalised Dutch VET system. In such systems, formal qualifications remain the fundamental ’currency’ for occupational access and employer screening, particularly at labour-market entry.

We classify respondents into five categories: (1) full match (reference), (2) vertical mismatch, (3) horizontal mismatch, (4) full mismatch (both vertical and horizontal), and (5) no paid work. These categories are mutually exclusive; the vertical and horizontal mismatch groups capture only one dimension of misalignment each, while the full mismatch category captures cases where both occur simultaneously. Vertical mismatch is identified from a question asking whether the educational level required by the employer for the job corresponds to the worker’s own level of education. Importantly, this measure reflects reported employer requirements rather than respondents’ subjective assessments of job complexity^4^. Individuals who report that the job requires a lower level of education than they possess are classified as having a vertical mismatch^5^. Horizontal mismatch is measured using respondents’ reports on whether their job requires the same field of study as their educational programme. Respondents indicating that their job requires an unrelated field, or no specific field, are categorised as horizontally mismatched. Full mismatch denotes cases where respondents simultaneously report a lower education requirement and a different (or no) field requirement.

#### Control variables

To reduce the risk of spurious associations, a set of covariates that are likely to influence both the probability of having an education-job mismatch and subsequent labour market outcomes are included in the model. These include age at labour market entry, gender, migration background (native vs. non-native Dutch), father’s education level (basic, vocational, university), and the level (2 to 4 years) and field of vocational education. The latter is categorised into four sectors: economics and administration, agriculture, technology, and health and welfare. We also include the final VET grade as a proxy for cognitive ability and account for job offers from training firms or, for school-based graduates, internship firms, differentiating between accepting, declining, or not receiving an offer. This is important because individuals who remain with their training firm are more likely to secure a job match, and work-based trainees are more likely to receive such offers and benefit from remaining with the firm (Bonnal et al. 2002).

Finally, while it is impossible to fully account for the potential selection into work-based or school-based training programmes, we address this by incorporating information on the local scarcity of training positions and the number of applications students submitted to secure a training position. The measure for the number of applications was standardised by comparing each student’s number of applications to the average number within their geographical region and field. A binary indicator was then created to denote whether a student had submitted a higher number of applications than the regional-field average. Furthermore, the mean number of applications per region and field was utilised as an indicator of the local scarcity of training positions, with higher values denoting greater shortages. Descriptive statistics are reported in Table 2 of the supplementary material.

While these measures help account for variation in training-market conditions and individual search effort, some relevant factors remain unobserved. In particular, the data do not contain information on employer-side characteristics such as firm-level hiring practices, vacancy structures, or occupational demand conditions, which may influence both the incidence of mismatch and subsequent career trajectories. Furthermore, our data do not contain information on non-cognitive skills, which have been shown to influence labour market outcomes through their effects on individual productivity (Heckman et al. 2006). Although we include a range of administrative and survey-based proxies to reduce the risk of omitted variable bias as comprehensively as the data allow, we cannot eliminate it entirely. Accordingly, while our longitudinal design and register-linked data provide a unique and detailed account of early career pathways, the identified mismatch patterns should be interpreted as descriptive associations reflecting selection and equilibrium labour-market sorting rather than isolated causal effects.

### Mixture Hidden Markov Models for sequence data

To examine how education-job mismatch relates to graduates’ early career trajectories, we model transitions between latent states of EQ using a Mixture Hidden Markov Model (MHMM). MHMMs can be regarded as an extension of latent class models for the analysis of longitudinal change in a discrete latent variable, while simultaneously clustering individuals into distinct groups (*trajectories*) based on their longitudinal process. The probabilistic framework allows for the analysis of complex sequence data, accounting for both temporal dynamics and heterogeneity within each temporal state. In this study, latent EQ states are inferred from observed patterns across three indicators: employment status, income, and working hours.

To estimate employment trajectories, the model incorporates three components: namely, the measurement, structural, and mixture components. The *measurement* component specifies the relationship between the observed indicators and the latent EQ states in a manner analogous to latent class analysis. Substantively, this part of the model determines how combinations of employment status, income and working hours correspond to particular states of EQ. The underlying assumption is that individuals belong to unobserved classes for which the observed data provide adequate information regarding class membership through a likelihood function, which seeks to identify the groups with the highest homogeneity within groups and highest heterogeneity between groups (Collins and Lanza 2009).

The *structural* component models the dynamics of movement between latent EQ states over time. This is achieved by estimating a series of multinomial logit models, estimating both the likelihood of an individual starting in each different EQ state at the first time point $t=0$ (initial state probabilities) and the likelihood of moving from a given state to another between consecutive time points (transition probabilities) (Vermunt et al. 2008). Transitions are assumed to follow a first-order Markov process, meaning that the state at time *t* depends only on the state at $t-1$, conditional on the realised sequence of prior states (Vermunt et al. 2008). The transition probabilities thus serve as an indication of patterns of stability or change that may be observed in the early stages of an individual’s career. To account for the dynamic and multifaceted nature of early careers, the standard assumption of time-homogeneous transitions is relaxed by allowing them to vary between months since graduation. This is implemented by including a time covariate in the transition probabilities.

Finally, individuals are grouped into discrete career trajectories, formally *mixtures*, based on their similarity in initial and transition probabilities. For each individual, the model estimates the probability of belonging to each trajectory. The analysis was conducted using Latent GOLD 6.0 (Vermunt and Magidson 2021).

As the MHMM is applied in an exploratory manner, a comprehensive interpretation of the results is necessary to derive the final model. In determining the number of latent states and trajectories, the optimal class solution is selected based on a combination of statistical and substantial criteria. Specifically, we prioritise solutions with the lowest values of the Akaike Information Criterion (AIC) and Bayesian Information Criterion (BIC) that also yield a substantively meaningful and stable class solution (Bacher and Vermunt 2010).

To identify the optimal number of EQ states, we first estimated a series of models ranging from 1 to 10 states. The model fit statistics (see Figure 1 in the supplementary material) show that both the AIC and BIC decrease as the number of classes increases. The model comprising eight discrete EQ states was identified as the one balancing a low value of the information criteria and demonstrating results that were theoretically meaningful and robust. Following the two-step procedure of Bakk and Kuha (2018), we first estimated the measurement model and subsequently fixed the measurement parameters when estimating the structural component. In the structural part, we estimated a set of models with varying numbers of mixtures. The information criteria achieved their minimum at five mixtures (see Figure 2 in the supplementary material), supporting the identification of five distinct early-career pathways.

Following the identification of these five distinct career trajectories, the analysis proceeds by treating the *predicted trajectory membership* as the dependent variable to assess the relationship between education–job mismatch and subsequent longitudinal career development^6^. A multinomial logistic regression model is used to estimate the probability of membership in each of the identified early career trajectories for graduates experiencing vertical, horizontal or full mismatches. We further include an interaction term to differentiate between school-based and work-based tracks. In light of the difficulties associated with interpreting coefficients from non-linear models, we present the results as Average Marginal Effects (AME), which represent the change in the probability of a given outcome in percentage points. Standard errors for the AME are computed using non-parametric bootstrap resampling (500 replications).

## Results

### States

The analysis identified eight distinct EQ states. Employment, income, and working hours contributed almost equally to differentiating the latent states, as indicated by factor loadings^7^ of 0.8 for employment and working hours and 0.9 for income. The results are presented in Table 1.

**Table 1:** Latent states of employment quality (in percentages)

	1 Affluent Stable Earners	2 Prosperous Stability	3 Stable Part-time	4 Flexible High- Earners	5 Mid-range Flexi- Earners	6 Flexible Part- time	7 Irregular workers	8 Non-employed individuals	Overall
% sample assigned	13.4	11.2	15.5	5.2	15.6	19.6	13.7	5.9	100
*Employment*									
Permanent	**87.7**	**100**	**100**	0.1	0.0	0.1	0.2	0.0	42.5
Fixed-term	0.0	0.0	0.0	**84.7**	**86.4**	**83.3**	0.5	0.0	34.3
TAW	0.0	0.0	0.0	**11.1**	**13.5**	**16.5**	**30.2**	0.0	6.0
On-call	0.0	0.0	0.0	4.1	0.1	0.1	**45.9**	0.0	6.5
Self-employment	**12.3**	0.0	0.0	0.0	0.0	0.0	**23.2**	2.1	4.9
No employment	0.0	0.0	0.0	0.0	0.0	0.0	0.0	**97.9**	5.8
*Income*									
0	0.0	0.0	0.0	0.0	0.0	0.0	0.0	**57.4**	3.4
1–1278	0.0	0.0	0.1	0.1	0.1	**46.9**	**61.8**	**30.5**	19.4
1279-1766	0.0	0.0	**40.0**	0.1	0.3	**50.8**	**20.6**	8.2	19.5
1767-2155	0.0	0.1	**59.6**	0.0	**53.9**	1.2	9.8	1.8	19.5
2156-2646	0.1	**99.7**	0.1	0.4	**44.2**	0.5	7.3	1.3	19.4
>2646	**99.9**	0.2	0.2	**99.4**	1.5	0.5	0.6	0.8	18.8
*Working hours*									
Zero hours	0.0	0.0	0.0	0.0	0.0	0.0	0.0	**100**	5.9
<12 hours	0.4	0.1	0.1	0.3	0.5	9.9	**21.1**	0.0	5.0
12-<20 hours	0.7	0.3	1.4	0.7	1.3	13.4	**24.5**	0.0	6.6
20–35 hours	**18.8**	**37.6**	**59.4**	15.0	**28.4**	**48.2**	**39.6**	0.0	36.0
>35 hours	**80.1**	**62.0**	**39.2**	**84.1**	**69.8**	**28.4**	14.8	0.0	46.5

The labelling of these states is based on a subjective assessment of their main characteristics in relation to the observed indicators of each state. The principal characteristics of each state are highlighted in bold. Source: SSD, 2015-2020, own estimation

*Affluent Stable Earners* (13.4%) represent the most secure group, characterised by permanent employment (87.7%), full-time work, and earnings above 2,646 euros. *Prosperous Stability* (11.2%) similarly combines permanent employment with moderately high income levels and predominantly full-time hours. Together, these states reflect strong labour-market attachment and high job security. *Stable Part-timers* (15.5%) also hold permanent contracts, but work fewer hours (mostly 20–35 per week) and earn low to moderate incomes (€1,279–€2,155), capturing stable employment conditions alongside part-time work schedules.

*Flexible High-Earners* (5.2%) are characterised by very high earnings (99.4% above €2,646) despite predominantly holding fixed-term (84.7%) or temporary-agency contracts (11.1%). They typically work full-time, illustrating that high incomes can coexist with contract instability. *Mid-range Flexi-Earners* (15.6%) display similar contractual flexibility (86.4% fixed-term) but at moderate income levels. *Flexible Part-timers* (19.6%) work part-time (20–35 hours) under mostly fixed-term contracts (83.3%) and typically earn €1,279–€1,766, reflecting more precarious conditions. *Irregular Workers* (13.7%) experience the greatest instability, with a considerable proportion in on-call (45.9%) or temporary agency work (30.2%). Most earn between 1 and 1,278 euros and work limited hours, reflecting the precarious conditions under which they operate. Finally, the *Non-employed Individuals* cluster (5.9%) consists almost entirely of individuals not engaged in any form of paid employment (97.9%), reporting no working hours and no income.

### Early career patterns of vocational graduates

**Fig. 1:**
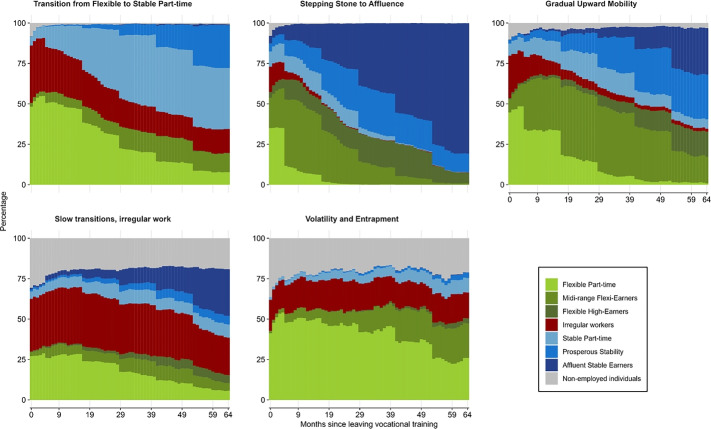
Status proportion plots of school-to-work patterns for vocational graduates in the Netherlands. Source: Statistics Netherlands, own calculations

Figure 1 presents the five distinct career trajectories identified by estimating transition probabilities between the latent EQ states over the five-year observation period. The first trajectory, *Transition from Flexible to Stable Part-time*, encompasses 29% of the sample and is characterised by an initial entry into the labour market through flexible part-time contracts, with nearly half of individuals starting in this state, while a notable share also enters in irregular work. Individuals in this trajectory gradually transition to more secure employment. At the end of the five-year period, 37.5% have transitioned to permanent part-time positions, and 25% have achieved prosperous stability. Nevertheless, part-time work remains the dominant pattern. This reflects a distinct feature of the Dutch labour market, where part-time employment is common even among entry workers. Women and healthcare workers, groups traditionally overrepresented in part-time work, dominate this trajectory.

The second trajectory, *Stepping Stone to Affluence* (25%) exemplifies a clear upward progression from temporary employment to *Affluent Stable Earners*. The majority of individuals (73%) enter the labour market in temporary roles, but transition relatively quickly into permanent, high-income positions. By the end of the observation period, the majority of individuals have attained the highest EQ state observed. This trajectory illustrates a successful school-to-work transition in which initial job insecurity serves as a stepping stone to stable and affluent employment, demonstrating substantial upward mobility from initially insecure positions.

The third trajectory, *Gradual Upward Mobility* (19%), represents a similar upward progression to the previous trajectory, but at a more gradual pace and with a greater prevalence of temporary contracts throughout the period. The trajectory includes a considerable proportion of *Mid-range Flexi-Earners*, indicating that while individuals experience upward mobility, a significant number remain in flexible and less secure forms of employment. This pattern indicates that for some individuals, the transition to stable, high-paying employment is more gradual and less complete, with flexible contracts persisting even as income levels increase.

The fourth trajectory, *Slow transitions, irregular work* (15%), represents a highly mixed pattern of career progression, with many individuals experiencing irregular employment and periods of non-employment. Transition probabilities are generally low, indicating limited movement out of precarious conditions. Although some individuals are able to transition into stable, higher-paying roles by the end of the observation period (28% succeed in becoming *Affluent Stable Earners*), 23% remain in irregular jobs, and 20% are non-employed, suggesting that delayed transitions constrain longer-term career stability and earnings. Given the heterogeneous nature of this trajectory, we conducted further analysis (Table 3, supplementary material) to examine differences between school-based and work-based graduates. Although the incidence of non-employment and stable part-time work is comparable across both groups, work-based graduates are 25 percentage points more likely to transition into *Affluent Stable Earners* and 11 percentage points less likely to remain *Irregular Workers* than their school-based peers.

The final and smallest trajectory, *Volatility and Entrapment* (12%) is characterised by persistent labour-market instability. Individuals frequently alternate between flexible part-time jobs, irregular work, and periods of non-employment, with only a small proportion progressing to permanent or higher-paying positions. The prevalence of flexible part-time employment throughout the observation period indicates that for a considerable proportion of this cohort, early career progression is a significant challenge. The prolonged reliance on insecure employment results in persistently low income and weak labour-market attachment, thereby reinforcing a cycle of instability. This trajectory reflects an unsuccessful school-to-work transition, characterised by persistent employment insecurity and constrained prospects for career advancement and financial stability.

### Education–job mismatch by vocational track

Prior to analysing how education–job mismatches relate to career trajectories, we estimate a multinomial logit model to test whether work-based graduates are less likely to experience mismatches, as posited in H2. The results are presented in Table 2.

**Table 2:** Average marginal effects from multinomial regression: mismatch

	No Mismatch	No Work	Full mismatch	Vertical Mismatch	Horizontal Mismatch
	AME	AME	AME	AME	AME
Work-based	0.099***	−0.031***	−0.068***	0.031***	−0.031***
	(0.01)	(0.00)	(0.01)	(0.01)	(0.01)
Sector (ref: Economics and administration)					
Agriculture	0.039	−0.012	−0.018	0.018	−0.027
	(0.02)	(0.01)	(0.02)	(0.01)	(0.02)
Technology	0.096***	−0.020***	−0.044***	0.021**	−0.053***
	(0.01)	(0.01)	(0.01)	(0.01)	(0.01)
Health and Welfare	0.262***	−0.030***	−0.112***	−0.027***	−0.094***
	(0.01)	(0.01)	(0.01)	(0.01)	(0.01)
MBO Level (ref: 2 years)					
3 years	0.111***	−0.044***	−0.054***	0.044***	−0.057***
	(0.02)	(0.01)	(0.01)	(0.01)	(0.01)
4 years	0.238***	−0.045***	−0.123***	−0.008	−0.063***
	(0.01)	(0.01)	(0.01)	(0.01)	(0.01)
AIC	23414.94				
BIC	23791.74				
N	10,364				

Model is adjusted for age at labour market entry, sex, migration background, and father’s education. Standard errors in parentheses are obtained using non-parametric bootstrap resampling (500 replications). Source: SVO 2016 and SSD, 2015-2020. *** p<0.01, ** p<0.05, * p<0.1

As expected, workplace-based graduates are less likely to experience education-job mismatches compared to their school-based counterparts. Specifically, work-based graduates show a 10 percentage points reduction in the probability of encountering any mismatch early in their careers. However, this reduction varies by mismatch type. While work-based graduates are less likely to encounter horizontal or full mismatches, they are somewhat more likely to experience vertical mismatches.

### Education–job mismatch and career trajectory membership

Table 3 presents the Average Marginal Effects (AME) estimating the association between education–job mismatches and the five identified career trajectories.

**Table 3:** Average marginal effects from multinomial regression: trajectory membership

	1 Flexible to stable part-time	2 Stepping stone to Affluence	3 Gradual upward mobility	4 Slow transitions, irregular work	5 Volatility & Entrapment
	AME	AME	AME	AME	AME
Work-based	0.043***	−0.014	−0.007	−0.018**	−0.005
	(0.01)	(0.01)	(0.01)	(0.01)	(0.01)
Mismatch					
*Reference: No mismatch*					
No work	−0.250***	−0.264***	−0.061**	0.261***	0.314***
	(0.02)	(0.01)	(0.02)	(0.03)	(0.02)
Horizontal mismatch	0.005	−0.036**	−0.028**	0.013	0.046***
	(0.01)	(0.01)	(0.01)	(0.01)	(0.01)
Vertical mismatch	0.002	−0.026	−0.067***	0.049**	0.042**
	(0.02)	(0.02)	(0.02)	(0.02)	(0.01)
Full mismatch	0.046***	−0.129***	−0.044***	0.043***	0.083***
	(0.01)	(0.01)	(0.01)	(0.01)	(0.01)
Work-based x Mismatch					
*Reference: No mismatch*					
No work	−0.430***	−0.195***	0.044	0.390***	0.192***
	(0.07)	(0.02)	(0.06)	(0.08)	(0.06)
Horizontal mismatch	−0.012	0.027	−0.029	−0.001	0.013
	(0.03)	(0.03)	(0.02)	(0.01)	(0.01)
Vertical mismatch	−0.038	−0.006	−0.018	0.031	0.019
	(0.04)	(0.03)	(0.02)	(0.02)	(0.01)
Full mismatch	0.046	−0.044	−0.050**	0.013	0.035***
	(0.03)	(0.02)	(0.02)	(0.02)	(0.01)
School-based x Mismatch					
*Reference: No mismatch*					
No work	−0.214***	−0.262***	−0.035	0.252***	0.278***
	(0.03)	(0.02)	(0.02)	(0.03)	(0.03)
Horizontal mismatch	0.040**	−0.061***	−0.019*	0.014	0.026***
	(0.02)	(0.01)	(0.01)	(0.01)	(0.01)
Vertical mismatch	0.042	−0.038	−0.064***	0.038***	0.023**
	(0.03)	(0.02)	(0.02)	(0.01)	(0.02)
Full mismatch	0.118***	−0.158***	−0.038**	0.034***	0.045***
	(0.02)	(0.01)	(0.01)	(0.01)	(0.01)
AIC	27336.73				
BIC	28090.15				
N	10,364				

Model is adjusted for field of education, MBO Level, age at labour market entry, sex, migration background, father’s education, final VET grade, number of applications, labour market conditions and whether the apprentice received a job offer from the training firm. Standard errors in parentheses are obtained using non-parametric bootstrap resampling (500 replications). The full model is available in the supplementary material. Source: SVO 2016 and SSD, 2015-2020.*** p<0.001, ** p<0.05, * p<0.01

Having a mismatch is associated with a significantly higher probability of following the *Volatility and Entrapment* trajectory, thereby providing support for H1a. For individuals experiencing a mismatch with regard to both field and level of education, the probability of belonging to this trajectory is 8.3 percentage points higher, while a single horizontal or vertical mismatch is associated with probabilities that are 4.6 and 4.2 percentage points higher, respectively. This finding indicates that mismatches, particularly combined ones, are linked to a higher likelihood of unstable early career trajectories characterised by frequent job transitions, temporary contracts, and unemployment, lending support for H1b. At the same time, mismatches are associated with a lower likelihood of entering more prosperous career trajectories. For instance, a full mismatch is associated with a 12.9 percentage points lower probability of following the *Stepping Stone to Affluence* trajectory. Single mismatches, whether horizontal or vertical, are also linked to a lower probability of following upward career paths, although these associations are smaller in magnitude. It is noteworthy that the identified negative pattern does not extend to the *Transition from Flexible to Stable Part-time* trajectory. Graduates experiencing a full mismatch are somewhat more likely to follow this path (4.6 percentage points), whereas a single mismatch is not significantly associated with following this trajectory. Although individuals in this trajectory predominantly enter the labour market in precarious positions, typically flexible part-time or irregular work, many subsequently transition into stable employment. Within the Dutch institutional context, where permanent part-time work is widespread and especially concentrated in sectors such as healthcare, this pathway can be regarded as a pragmatic solution to labour-market stability for graduates encountering a full mismatch. Furthermore, while unemployment is an integral part of the trajectory and must be interpreted with caution, early-career joblessness is associated with a 31.4 percentage points higher probability of following the *Volatile and Entrapment* trajectory and a 26.4 percentage points lower likelihood of entering the *Stepping Stone to Affluence* trajectory.

Next, we examine whether the negative associations of job mismatches vary between work-based and school-based graduates. The results (Table 3) reveal a notable contrast between the two groups. Contrary to our expectations in H3, school-based graduates face more pronounced disadvantages related to mismatches compared to their work-based counterparts. In terms of trajectory membership, differences between the two vocational tracks are rather small. Work-based graduates are slightly more likely to follow the trajectory *Transition from Flexible to Stable Part-time*. In contrast, the negative associations between job mismatches and trajectory membership are more pronounced for school-based graduates than for their work-based counterparts. For school-based graduates, a full mismatch is associated with a 15.8 percentage points lower likelihood of entering the *Stepping Stone to Affluence* trajectory, and a 4.5 percentage points higher probability of being observed in the *Volatile and Entrapment* trajectory. A vertical mismatch is associated with a 6.4 percentage points lower probability of being observed in the *Gradual Upward Mobility* trajectory, while a horizontal mismatch relates to a 6.1 percentage points lower probability of following the *Stepping Stone to Affluence* trajectory. In contrast, for work-based graduates, single mismatches, whether horizontal or vertical, are not significantly associated with trajectory membership. However, a full mismatch is associated with a 3.5 percentage point higher likelihood of being observed in the *Volatile and Entrapment* trajectory, while it is linked to a 5 percentage points lower probability of entering the trajectory characterised by gradual upward mobility.

Furthermore, across both groups, joblessness is most notably associated with a diminished likelihood of entering the *Stepping Stone to Affluence* trajectory. Specifically, school-based graduates who are initially unemployed have a 27.8 percentage points higher probability of being observed in the *Volatility and Entrapment* trajectory compared to those in matched positions, while for work-based graduates, this probability is 19.2 percentage points higher. However, work-based graduates show a 39 percentage points higher probability of being observed in the *Slow transitions, irregular work* trajectory when starting unemployed. In essence, the results suggest that while unemployment for work-based graduates is primarily linked to patterns of limited upward mobility, school-based graduates demonstrate a stronger association with trajectories of persistent volatility and entrapment.

### Robustness checks

To assess the sensitivity of our findings to measurement timing and latent classification uncertainty, we conducted two additional robustness checks. First, because the SVO was administered approximately one year after graduation, the observed mismatch captures the employment situation at the survey date, which may not always correspond to the first job for graduates who experienced early job changes. To address this, we re-estimated our models on a restricted sample of individuals whose job at the time of the survey corresponds to their first observed employment spell after graduation. This was determined by cross-referencing survey-reported job start dates with individual-level administrative records of employment spells. Excluding respondents who had changed jobs prior to the survey interview reduces the analytical sample to 8,108 individuals. The results remain substantively unchanged in terms of direction, magnitude, and statistical significance (see Table 6 in the supplementary materials), indicating that the observed associations between mismatch and career trajectories are not driven by measurement differences arising from job changes occurring prior to the survey interview.

Second, because trajectory membership is derived from a MHMM, classification uncertainty may affect the estimation of the relationship between mismatch and trajectory membership. To address this, we re-estimated the relationship between covariates and trajectory membership using the bias-adjusted three-step approach implemented in Latent GOLD, which explicitly accounts for classification uncertainty in the latent class assignment (Bakk and Kuha 2021). The estimated associations are very similar in direction and magnitude to those obtained from the multinomial regression based on modal trajectory assignment, indicating that the conclusions are not sensitive to potential classification error. The multinomial regression results are reported in the main text because they can be readily translated into AME, which provide a more intuitive interpretation of differences in predicted trajectory probabilities. The Latent GOLD output of the three-step approach is reported in the online supplementary material^8^.

## Discussion and conclusion

This paper examines how education–job mismatches at labour market entry are associated with early career trajectories of vocational graduates from school- and work-based programmes in the Netherlands. Using longitudinal register data and a Mixture Hidden Markov Model, we identify distinct patterns of employment quality over the first five years after graduation. A central contribution of this research is to provide a dynamic perspective on how education–job mismatches relate to subsequent patterns of labour-market integration and career progression by adopting a multidimensional trajectory approach that simultaneously captures employment stability, contract type, working hours, and wages.

The results reveal substantial heterogeneity in early transitions from education to work. While some graduates progress towards secure and well-remunerated employment, others experience volatile career paths marked by temporary contracts, frequent job changes, and limited upward mobility. Temporary employment has become a persistent condition for many graduates, reflecting the increasingly diverse nature of school-to-work transitions. These deviations from more traditional VET pathways are associated with more challenging patterns of labour market integration and correlate with more constrained career progression.

In accordance with assignment theory, our findings demonstrate that education-job mismatches at labour-market entry are associated with vocational graduates’ early careers. Frequently, vertical and horizontal mismatches coincide, and their combined occurrence is associated with particularly disadvantageous outcomes. Graduates experiencing a full mismatch are less likely to be observed in career trajectories involving upward mobility. Concurrently, the probability of experiencing a volatile and precarious career trajectory, characterised by temporary employment, job instability, and periods of non-employment, is significantly higher. The negative associations of a combined mismatch appear to be more pronounced, as such situations correlate with lower applicability of acquired skills and stronger negative signals sent to future employers. This finding is consistent with statistical discrimination by employers (Altonji and Pierret 2001), who may interpret a mismatch as an observable signal of lower productivity or adaptability when hiring young workers.

Furthermore, the strong association of a dual mismatch with the *Volatility & Entrapment* trajectory is consistent with the dynamic sorting mechanisms proposed by Gibbons and Waldman (1999), where workers who fail to signal high ability early on are less likely to access fast-track promotions or wage growth. In our study, a dual mismatch at entry may function as an initial signal that is associated with a serial correlation of disadvantageous outcomes. This suggests that the persistent longitudinal patterns often attributed to mismatch may reflect the results of repeated sorting by employers who use early-career job allocations as proxies for worker productivity within an equilibrium matching process. Together, these mechanisms suggest that early mismatches not only reflect initial allocation difficulties but are also consistent with persistent vocational disadvantages that extend beyond entry.

However, our findings also reveal important heterogeneity, providing partial support for the career mobility perspective which highlights individual adaptation. A proportion of mismatched graduates are more likely to being observed in the *Transition from Flexible to Stable Part-time* trajectory. Although this pathway begins in rather precarious employment, a significant proportion of individuals subsequently transition into permanent positions, albeit predominantly part-time. This suggests that for some graduates, employment in mismatched positions may represent a stepping stone or adaptive pattern toward labour-market attachment and stability. A possible explanation relates to sectoral demand: in sectors such as health and social care, where there is a persistent shortage of labour, and part-time work is a common feature of institutional practice, employers may recruit graduates whose qualifications do not fully align with the role, but who can nevertheless perform entry-level or support roles. For a subset of mismatched graduates, such jobs may therefore serve as a pragmatic route into the labour market, facilitating initial attachment and gradual stabilisation, even if longer term prospects for wage progression are more limited. This finding suggests that mismatch is not uniformly linked to instability but may, under specific institutional conditions, reflect adaptive matching that channels graduates into stable yet lower-intensity employment trajectories, rather than resulting in outright precariousness.

With regard to the different training programmes, our findings indicate a weaker link between single mismatches and career instability for workplace-based graduates compared to their school-based counterparts. This is likely because work-based training typically strengthens occupation-specific skills and cultivates a broader set of transferable competencies such as familiarity with workplace routines, collaboration, and discipline, that are valued by employers across sectors. Consequently, for these graduates, a single mismatch is not associated with a negative signal strong enough to be linked to poorer labour market integration. In contrast, school-based graduates demonstrate more pronounced disadvantages when experiencing a mismatch. The higher prevalence of mismatches among this group, together with more pronounced disadvantages for career progression, suggests that weaker employer linkages and less work experience are associated with a stronger relationship between mismatch and career progression for school-based graduates. For these graduates, horizontal mismatches in particular are associated with a lower likelihood of entering prosperous stepping-stone trajectories and a higher probability of unstable career paths.

However, the vocational resilience associated with work-based training appears to have clear limits. When graduates experience a dual mismatch, the typical advantages of work-based training are substantially attenuated. In such cases, prior work experience no longer appears to mitigate the disadvantages associated with the simultaneous loss of skill relevance and negative signalling–factors that are linked to more constrained upward mobility and limited access to high-quality employment. Taken together, these findings suggest that within occupational systems, dual mismatch is associated with substantial labour-market disadvantages, whereby credential–job alignment may operate as an important sorting mechanism linked to career progression.

Finally, unemployment at labour market entry is associated with a significantly higher probability of following unstable careers characterised by underemployment and temporary work. This finding aligns with previous research, identifying unemployment as one of the most disruptive early-career events (Gangl 2004), particularly for vocational graduates, whose occupation-specific skills may become redundant if they are not utilised.

A few limitations warrant consideration. First, information on education–job mismatch is only available at labour market entry and is therefore treated as time-invariant. While this approach captures the initial allocation into employment, a crucial moment in shaping early trajectories, it does not allow us to observe whether match status subsequently improves or deteriorates. Given the high job mobility typically observed in the first years after graduation, changes in match quality are likely; however, the data do not provide repeated measures of mismatch, limiting our ability to model such dynamics. Second, although we do not assume a VET-track preference, selection into work-based or school-based pathways is non-random and may be influenced by both observable and unobservable factors. Although we control for a comprehensive set of potential confounders, unobserved characteristics may still influence programme choice and cannot be fully captured in the analysis. Moreover, the data do not contain information on non-cognitive skills, local labour-market demand conditions or firm-level hiring practices, which may influence both the incidence of education–job mismatch and subsequent career trajectories. Finally, our analysis focuses on vocational graduates who transition directly into the labour market. As a considerable proportion of graduates pursue further education, future research could explore how further training acts as a mechanism for correcting initial mismatch experiences and subsequent career progression.

## Supplementary Information

Supplementary file 1

## Data Availability

This study uses administrative microdata provided by Statistics Netherlands (CBS). Due to legal and privacy restrictions, these data cannot be shared publicly. Researchers may apply for access to CBS microdata through the CBS microdata portal. Further information on application procedures and access conditions is available at the Statistics Netherlands website:https://www.cbs.nl/en-gb/our-services/customised-services-microdata/microdata-conducting-your-own-research. The analytical code used in this study is available from the authors upon request. Access to the code may require correspondence to ensure compatibility with CBS regulations.

## Footnotes

1 Some employers offer work-based vocational training as (formal) further training for employees with lower qualifications in order to advance in their careers. This is also reflected in the higher average age of those participating in work-based compared to school-based training.

2 This group comprises 74 cases.

3 This includes 2.8% of individuals over the study period.

4 In the Dutch vocational labour market, educational requirements are typically explicitly stated in vacancies and recruitment procedures, as occupations linked to MBO qualifications are highly standardised and regulated. Because respondents report the employer’s requirement rather than their own assessment, systematic reporting bias is less likely in this institutional context.

5 In our context, undereducated cases are extremely rare and are grouped with adequately matched workers.

6 The resulting model demonstrates almost perfect individual accuracy in classifying states (entropy of 0.99) and relatively high accuracy in classifying trajectories (entropy of 0.7). This justifies the use of modal trajectory assignment–assigning individuals to the trajectory with the highest posterior classification probability–as the basis for our subsequent regression analysis.

7 The factor loading indicates the strength of the factor predicting the item.

8 Supplementary materials are available at https://osf.io/vj942.
