# Supplementary material for: Job mismatches and their implications for the career development of vocational graduates
Source: Empir Res Vocat Educ Train. 2026 Apr 22;18(1):5. doi: 10.1186/s40461-026-00207-w (PMC13102878; doi:10.1186/s40461-026-00207-w)
Supplement: Supplementary file 1 [file 40461_2026_207_MOESM1_ESM.pdf]

Online Supplementary Materials

Job mismatches and their implications for the career development of vocational graduates

Author
Affiliation

Contents

Table 1

Analytic sample restrictions and the associated changes in case numbers

**Criteria**	**N**	**%**
Initial sample	37,252	100
Exclude part-time education	37,066	99
Exclude MBO Level 1	35,539	95
Age restriction (28 years)	31,701	85
Exclude continued education	13,901	37
Match survey with register data	10,934	29
Exclude moved abroad	10,637	29
Exclude education within 6 months	10,629	29
Exclude never employed/self-employed	10,555	28
Listwise deletion of missing values	10,364	28

Source: SVO 2016 and SSD, 2015-2020, own calculations.

Table 2

Descriptive Statistics

	Workplace-based		School-based		Total	
	N	%	N	%	N	%
Mismatch						
No mismatch	1,562	65.1	4,503	57.4	6,065	58.5
Horizontal mismatch	343	13.6	1,235	15.7	1,578	15.2
Vertical mismatch	254	10.1	374	4.8	628	6.1
Full mismatch	307	12.2	1,361	17.3	1,668	16.1
No work	51	2.0	374	4.8	425	4.1
Level of diploma						
2 years	563	22.3	1,008	12.9	1,571	15.2
3 years	1,187	47.2	1,821	23.2	3,008	29
4 years	767	30.5	5,018	63.9	5,785	55.8
Field of diploma						
Economics and business	688	27.3	2,338	29.8	3,026	29.2
Agriculture	149	5.9	552	7.0	701	6.8
Technology	950	37.8	1,833	23.4	2,783	26.8
Health and Welfare	730	29	3,124	39.8	3,854	37.2
Job offer						
yes, accepted	1,845	73.3	3,528	44.9	5,373	51.8
yes, rejected	279	11.1	1,417	18.1	1,696	16.4
no offer	393	15.6	2,902	37.0	3,295	31.8
Number of applications submitted						
More than average	600	23.8	2,888	36.8	3,488	33.6
Less than average	1,917	76.2	4,959	63.2	6,876	66.4
Gender						
Female	1,070	42.5	4,640	59.1	5,710	55.1
Male	1,447	57.5	3,207	40.9	4,654	44.9
Migrarion background						
Native	2,300	91.4	6,687	85.2	8,987	86.7
Non-native	217	8.6	1,160	14.8	1,377	13.3
Age	10,364	22.2 (2.6)	10,364	20.9 (1.8)	10,364	21.2 (2.1)
Training supply	10,364	1.6 (0.2)	10,364	1.6 (0.3)	10,364	1.6 (0.3)
Father's education						
Basic education	626	24.8	1,933	24.6	2,559	24.7
Vocational education	888	35.3	2,559	32.6	3,447	33.3
University	495	19.7	1,817	23.2	2,312	22.3
Don't know	508	20.2	1,538	19.6	2,046	19.7
Total	2,517	24.3	7,847	75.7	10,364	100

Source: SVO 2016 and SSD, 2015-2020, own calculations.

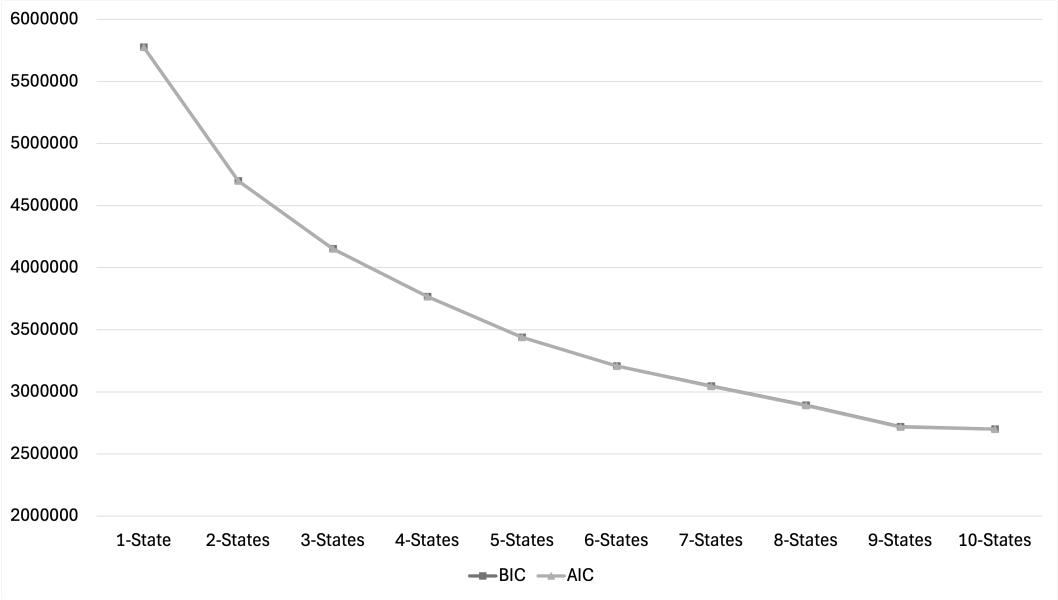

Figure 1

BIC and AIC for different state solutions. Source: SVO 2016 and SSD, 2015-2020, own calculations.

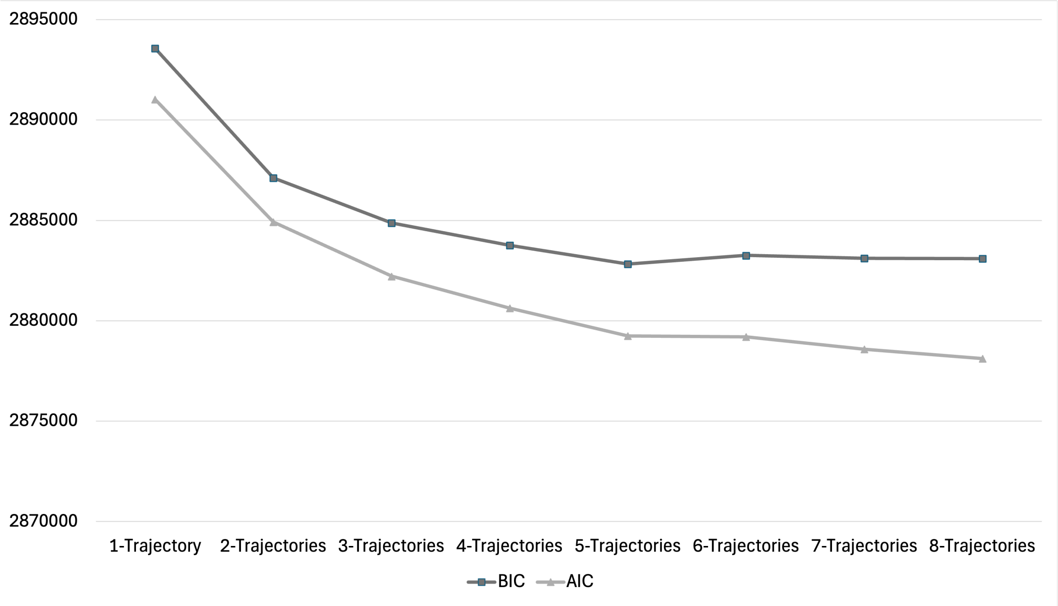

Figure 2

BIC and AIC for different trajectory solutions. Source: SVO 2016 and SSD, 2015-2020, own calculations.

Table 3

Average Marginal Effects (AME) from multinomial regression with last state within trajectory 4 as DV (in percentage points)

	Affluent Stable Earners	Prosperous Stability	Stable Part-Time	Flexible High Earners	Mid-range Flexi-Earners	Flexible Part-Time	Irregular Workers	Non-employed Individuals
**Work-based**	0.25*** (0.04)	-0.05*** (0.01)	0.01*** (0.03)	0.00 (0.02)	0.02*** (0.02)	-0.05*** (0.01)	-0.11*** (0.03)	-0.01 (0.03)
**Control variables**	yes							
**AIC**	4183.26							
**N**	10,364							

Note: Standard errors in parentheses are obtained using non-parametric bootstrap resampling (500 replications). Source: SVO 2016 and SSD, 2015-2020, own calculations.
*** $p < 0.001$, ** $p < 0.01$, * $p < 0.05$. *Additional explanation:* This table presents the AME from a multinomial regression analysis, where the dependent variable is the last state within the trajectory *Slow transitions, irregular work*. We conducted this additional analysis to address the heterogeneity in the final states observed in this trajectory, and to examine whether the probability of ending in these states varied by the type of vocational training. The results indicate notable differences for the states *Affluent Stable Earners* and *Irregular workers*. In contrast, similar analyses for the other trajectories did not reveal a similar variation between the final state and the vocational track.

Table 4

Average Marginal Effects (AME) from multinomial regression with Education-job mismatch as DV (in percentage points)

	No mismatch	No Work	Full mismatch	Vertical mismatch	Horizontal mismatch
**Work-based**	9.9***<br>(0.01)	-3.1***<br>(0.00)	-6.8***<br>(0.01)	3.1***<br>(0.01)	-3.1***<br>(0.01)
**Sector**					
Reference: Economics and administration					
Agriculture	3.9<br>(0.02)	-1.2<br>(0.01)	-1.8<br>(0.02)	1.8<br>(0.01)	-2.7<br>(0.02)
Technology	9.6***<br>(0.01)	-2.0***<br>(0.01)	-4.4***<br>(0.01)	2.1**<br>(0.01)	-5.3***<br>(0.01)
Health & Welfare	26.2***<br>(0.01)	-3.0***<br>(0.01)	-11.2***<br>(0.01)	-2.7***<br>(0.01)	-9.4***<br>(0.01)
**MBO Level**					
Reference: 2 years					
3 years	11.1***<br>(0.02)	-4.4***<br>(0.01)	-5.4***<br>(0.01)	4.4***<br>(0.01)	-5.7***<br>(0.01)
4 years	23.8***<br>(0.01)	-4.5***<br>(0.01)	-12.3***<br>(0.01)	-0.8<br>(0.01)	-6.3***<br>(0.01)
**Female**	-4.1***<br>(0.01)	1.5***<br>(0.00)	3.1***<br>(0.01)	-0.4<br>(0.01)	0.0<br>(0.01)
**Age**	0.6***<br>(0.00)	0.4***<br>(0.00)	-0.8***<br>(0.00)	-0.4**<br>(0.00)	0.1<br>(0.00)
**Native Dutch**	-5.5***<br>(0.01)	2.8***<br>(0.01)	1.6<br>(0.01)	-0.4<br>(0.01)	1.5<br>(0.01)
**Fathers education**					
Reference: Vocational education (MBO)					
Basic education	-1.9<br>(0.01)	-0.1<br>(0.00)	2.9**<br>(0.01)	1.1<br>(0.01)	-1.8*<br>(0.01)
University	-2.1<br>(0.01)	0.0<br>(0.01)	2.1*<br>(0.01)	-0.6<br>(0.01)	0.6<br>(0.01)
Don't know	-8.5***<br>(0.01)	1.7**<br>(0.01)	4.2***<br>(0.01)	0.2<br>(0.01)	2.4*<br>(0.01)
AIC	23414.94				
BIC	23791.74				
Log Likelihood	-11638.38				
N	10,364				

Source: SVO 2016 and SSD, 2015-2020, own calculations. Standard errors in parentheses are obtained using non-parametric bootstrap resampling (500 replications).
*** p<0.001, ** p<0.01, * p<0.05

Table 5

Average Marginal Effects (AME) from multinomial regression with Trajectory as DV (in percentage points)

	Flexible to Stable Part-time	Stepping-stone to Affluence	Gradual upward mobility	Slow transitions, Irregular work	Volatility and Entrapment
**Overall probability**	29.6	24.6	18.9	15.0	11.9
**Work-based**	4.3***<br>(0.01)	-1.4<br>(0.01)	-0.7<br>(0.01)	-1.8**<br>(0.01)	-0.5<br>(0.01)
**Mismatch**					
Reference: No mismatch					
No work	-25.0***<br>(0.02)	-26.4***<br>(0.01)	-6.1**<br>(0.02)	26.1***<br>(0.03)	31.4***<br>(0.02)
Horizontal mismatch	0.5<br>(0.01)	-3.6**<br>(0.01)	-2.8**<br>(0.01)	1.3<br>(0.01)	4.6***<br>(0.01)
Vertical mismatch	0.2<br>(0.02)	-2.6<br>(0.02)	-6.7***<br>(0.02)	4.9**<br>(0.02)	4.2**<br>(0.01)
Full mismatch	4.6***<br>(0.01)	-12.9***<br>(0.01)	-4.4***<br>(0.01)	4.3***<br>(0.01)	8.3***<br>(0.01)
**Work-based x Mismatch**					
Reference: No mismatch					
No work	-43.0***<br>(0.07)	-19.5***<br>(0.02)	-4.4<br>(0.06)	39.0***<br>(0.08)	19.2***<br>(0.06)
Horizontal mismatch	-1.2<br>(0.03)	2.7<br>(0.03)	-2.9<br>(0.02)	-0.1<br>(0.01)	1.3<br>(0.01)
Vertical mismatch	-3.8<br>(0.04)	-0.6<br>(0.03)	-1.8<br>(0.02)	3.1<br>(0.02)	1.9<br>(0.01)
Full mismatch	4.6<br>(0.03)	-4.4<br>(0.02)	-5.0**<br>(0.02)	1.3<br>(0.02)	3.5***<br>(0.01)
**School-based x Mismatch**					
Reference: No mismatch					
No work	-21.4***<br>(0.03)	-26.2***<br>(0.02)	-3.5***<br>(0.02)	25.1***<br>(0.03)	27.8***<br>(0.03)
Horizontal mismatch	4.0<br>(0.02)	-6.1***<br>(0.01)	-1.9<br>(0.01)	1.4<br>(0.01)	2.6***<br>(0.01)
Vertical mismatch	4.2<br>(0.03)	-3.8<br>(0.02)	-6.4***<br>(0.02)	3.8**<br>(0.01)	2.3**<br>(0.02)
Full mismatch	11.8***<br>(0.02)	-15.8***<br>(0.01)	-3.8**<br>(0.01)	3.4***<br>(0.01)	4.5***<br>(0.01)
**Level**					
Reference: 2 years of training					
3 Years	1.7<br>(0.02)	6.6***<br>(0.01)	-0.7<br>(0.01)	-3.9***<br>(0.01)	-3.8***<br>(0.01)
4 Years	-6.3***<br>(0.01)	14.7***<br>(0.01)	3.3**<br>(0.01)	-4.5***<br>(0.01)	-7.1***<br>(0.01)

Table 5 continued from previous page

	Flexible to Stable Part-time	Stepping-stone to Affluence	Gradual upward mobility	Slow transitions, Irregular work	Volatility and Entrapment
**Sector**					
Reference: Economics and administration					
Agriculture	6.9***	-6.4***	-5.5***	6.8***	-1.9
	(0.02)	(0.02)	(0.02)	(0.01)	(0.01)
Technology	-8.9***	11.5***	-2.8**	3.0***	-2.8**
	(0.01)	(0.02)	(0.01)	(0.01)	(0.01)
Health and welfare	7.3***	-1.4	-3.2**	1.5	-4.2***
	(0.02)	(0.01)	(0.01)	(0.01)	(0.01)
**Job offer from training company/internship**					
Reference: no offer					
Yes, accepted	2.2*	7.4***	-1.5	-2.8***	-5.2***
	(0.01)	(0.01)	(0.01)	(0.01)	(0.01)
Yes, rejected	-1.7	5.9***	-2.3*	0.5	-2.3**
	(0.01)	(0.01)	(0.01)	(0.01)	(0.01)
**Shortage of training places**	3.1	-11.5***	-2.4	5.7***	5.0***
	(0.02)	(0.02)	(0.02)	(0.01)	(0.01)
**Number of applications**	1.5	-6.7***	1.3	0.6	3.2***
	(0.01)	(0.01)	(0.01)	(0.01)	(0.01)
**Age**	-1.9***	1.1***	0.7***	0.1	0.0
	(0.00)	(0.00)	(0.00)	(0.01)	(0.00)
**Female**	23.9***	-24.0***	-4.9***	-0.4	5.4***
	(0.01)	(0.01)	(0.01)	(0.01)	(0.01)
**Father's education**					
Reference: Vocational training (MBO)					
Basic Education	-1.8	-0.8	0.0	2.2**	0.3
	(0.01)	(0.01)	(0.01)	(0.01)	(0.01)
University	0.1	-3.9***	1.0	0.6	2.2**
	(0.01)	(0.01)	(0.01)	(0.01)	(0.01)
Don't know	1.2	-3.2**	-0.1	1.2	1.0
	(0.01)	(0.01)	(0.01)	(0.01)	(0.01)
**Migration background**	-9.8***	-3.7**	4.3***	4.1***	5.0***
	(0.01)	(0.01)	(0.01)	(0.01)	(0.01)
**Final VET Grade**	-1.0	1.8***	-1.2***	0.7*	-0.3
	(0.00)	(0.00)	(0.00)	(0.00)	(0.00)
AIC	27336.73				
BIC	28090.15				
Log Likelihood	-13564.37				
McFadden R^2	0.14				
N	10,364				

Notes: Standard errors in parentheses are obtained using non-parametric bootstrap resampling (500 replications). *** $p < 0.001$, ** $p < 0.01$, * $p < 0.05$.

Table 6

Robustness check: Average marginal effects (AME) from multinomial regression using subsample where mismatch corresponds to first job after graduation

	Flexible to Stable Part-time	Stepping-stone to Affluence	Gradual upward mobility	Slow transitions, Irregular work	Volatility and Entrapment
**Overall probability**	29.6	24.6	18.9	15.0	11.9
**Work-based**	4.3***	0.3	-0.4	-2.7**	-1.5
	(0.01)	(0.01)	(0.01)	(0.01)	(0.01)
**Mismatch**					
Reference: No mismatch					
No work	-25.4***	-28.4***	-3.5	24.8***	32.5***
	(0.02)	(0.01)	(0.02)	(0.03)	(0.03)
Horizontal mismatch	0.6	-1.8*	-2.9**	0.9	3.2***
	(0.01)	(0.01)	(0.01)	(0.01)	(0.01)
Vertical mismatch	-0.2	-3.1	-5.2***	5.1**	3.4**
	(0.02)	(0.02)	(0.02)	(0.02)	(0.01)
Full mismatch	6.5***	-13.0***	-4.6***	5.1***	5.9***
	(0.01)	(0.01)	(0.01)	(0.01)	(0.01)
**Work-based x Mismatch**					
Reference: No mismatch					
No work	-42.3***	-22.1***	7.8	35.2***	21.5***
	(0.07)	(0.02)	(0.07)	(0.08)	(0.06)
Horizontal mismatch	-1.1	4.2	-2.5	-1.1	0.5
	(0.04)	(0.03)	(0.02)	(0.01)	(0.01)
Vertical mismatch	-4.0	-0.0	-0.4	3.0	1.4
	(0.04)	(0.03)	(0.03)	(0.02)	(0.01)
Full mismatch	5.4	-2.4	-5.0**	0.8	2.2*
	(0.04)	(0.03)	(0.02)	(0.02)	(0.01)
**School-based x Mismatch**					
Reference: No mismatch					
No work	-27.8***	-22.5***	-1.3	22.0***	29.6***
	(0.04)	(0.02)	(0.02)	(0.03)	(0.04)
Horizontal mismatch	3.3	-4.3**	-2.3	1.3	2.0**
	(0.02)	(0.02)	(0.01)	(0.01)	(0.01)
Vertical mismatch	3.9	-3.7	-5.7**	3.5**	2.0*
	(0.04)	(0.03)	(0.02)	(0.02)	(0.01)
Full mismatch	14.0***	-17.2***	-5.5**	3.7***	3.6***
	(0.02)	(0.01)	(0.01)	(0.01)	(0.01)
**Level**					
Reference: 2 years of training					
3 Years	1.4	7.0***	-0.7	-4.9***	-2.8***
	(0.02)	(0.01)	(0.01)	(0.01)	(0.01)
4 Years	-7.0***	15.7***	2.5	-5.5***	-5.8***
	(0.02)	(0.01)	(0.01)	(0.01)	(0.01)

Table 6 continued from previous page

	Flexible to Stable Part-time	Stepping-stone to Affluence	Gradual upward mobility	Slow transitions, Irregular work	Volatility and Entrapment
**Sector**					
Reference: Economics and administration					
Agriculture	5.7**	-7.9***	-4.2***	8.4***	-2.0
	(0.02)	(0.02)	(0.02)	(0.02)	(0.01)
Technology	-9.8***	11.1***	-2.2	3.0**	-2.1**
	(0.01)	(0.01)	(0.01)	(0.01)	(0.01)
Health and welfare	7.0***	-3.6*	-1.1	0.7	-3.1**
	(0.02)	(0.02)	(0.01)	(0.01)	(0.01)
**Job offer from training company/internship**					
Reference: no offer					
Yes, accepted	2.6*	7.3***	-1.7	-4.2***	-4.0***
	(0.01)	(0.01)	(0.01)	(0.01)	(0.01)
Yes, rejected	-2.0	5.6***	-2.7*	0.2	-1.0
	(0.01)	(0.01)	(0.01)	(0.01)	(0.01)
**Shortage of training places**	3.9	-13.0***	-0.9	5.4***	4.6***
	(0.02)	(0.02)	(0.02)	(0.02)	(0.01)
**Number of applications**	2.3*	-7.0***	1.1	1.1	2.5***
	(0.01)	(0.01)	(0.01)	(0.01)	(0.01)
**Age**	-1.6***	1.1***	0.5***	0.1	-0.2
	(0.00)	(0.00)	(0.00)	(0.00)	(0.00)
**Female**	24.6***	-23.9***	-4.0***	-0.6	3.9***
	(0.01)	(0.01)	(0.01)	(0.01)	(0.01)
**Father's education**					
Reference: Vocational training (MBO)					
Basic Education	-1.1	-1.2	-0.6	2.5**	0.4
	(0.01)	(0.01)	(0.01)	(0.01)	(0.01)
University	2.4	-5.0***	0.5	1.4	0.7
	(0.01)	(0.01)	(0.01)	(0.01)	(0.01)
Don't know	2.4	-3.9**	-0.3	1.4	0.4
	(0.01)	(0.01)	(0.01)	(0.01)	(0.01)
**Migration background**	-9.3***	-4.6***	5.4***	4.1***	4.4***
	(0.01)	(0.01)	(0.01)	(0.01)	(0.01)
**Final VET Grade**	-0.9	1.7***	-1.0**	0.6	-0.4
	(0.00)	(0.00)	(0.00)	(0.00)	(0.00)
AIC	21013.32				
BIC	21741.38				
Log Likelihood	-10402.66				
McFadden R^2	0.15				
N	8,108				

Notes: Standard errors in parentheses are obtained using non-parametric bootstrap resampling (500 replications). *** $p < 0.001$, ** $p < 0.01$, * $p < 0.05$.
